# Bivalent Llama Single-Domain Antibody Fragments against Tumor Necrosis Factor Have Picomolar Potencies due to Intramolecular Interactions

**DOI:** 10.3389/fimmu.2017.00867

**Published:** 2017-07-31

**Authors:** Els Beirnaert, Aline Desmyter, Silvia Spinelli, Marc Lauwereys, Lucien Aarden, Torsten Dreier, Remy Loris, Karen Silence, Caroline Pollet, Christian Cambillau, Hans de Haard

**Affiliations:** ^1^Ablynx NV, Ghent, Belgium; ^2^Architecture et Fonction des Macromolécules Biologiques, Aix-Marseille Université, Campus de Luminy, Marseille, France; ^3^Architecture et Fonction des Macromolécules Biologiques, Centre National de la Recherche Scientifique (CNRS), Campus de Luminy, Marseille, France; ^4^Department of Immunopathology, Sanquin Research, Amsterdam, Netherlands; ^5^Structural Biology Brussels, Vrije Universiteit Brussel, Brussels, Belgium; ^6^Structural Biology Research Center, VIB, Brussels, Belgium

**Keywords:** tumor necrosis factor, cytokine, inflammation, nanobody, VHH, intramolecular binding, crystal structure

## Abstract

The activity of tumor necrosis factor (TNF), a cytokine involved in inflammatory pathologies, can be inhibited by antibodies or trap molecules. Herein, llama-derived variable heavy-chain domains of heavy-chain antibody (VHH, also called Nanobodies™) were generated for the engineering of bivalent constructs, which antagonize the binding of TNF to its receptors with picomolar potencies. Three monomeric VHHs (VHH#1, VHH#2, and VHH#3) were characterized in detail and found to bind TNF with sub-nanomolar affinities. The crystal structures of the TNF–VHH complexes demonstrate that VHH#1 and VHH#2 share the same epitope, at the center of the interaction area of TNF with its TNFRs, while VHH#3 binds to a different, but partially overlapping epitope. These structures rationalize our results obtained with bivalent constructs in which two VHHs were coupled *via* linkers of different lengths. Contrary to conventional antibodies, these bivalent Nanobody™ constructs can bind to a single trimeric TNF, thus binding with avidity and blocking two of the three receptor binding sites in the cytokine. The different mode of binding to antigen and the engineering into bivalent constructs supports the design of highly potent VHH-based therapeutic entities.

## Introduction

Tumor necrosis factor (TNF) is a pleiotropic cytokine with beneficial functions in immune regulation and host defense, but deleterious pro-inflammatory and cytotoxic functions during inflammation. TNF is a pro-inflammatory cytokine that represents a critical mediator of the autoimmune process, playing a key role in several inflammatory diseases, including rheumatoid arthritis (RA), ulcerative colitis, and Crohn’s disease. Increased understanding of the biological basis of autoimmunity has led to its identification as a major regulator of immune homeostasis, permitting the development of new TNF antagonists at the forefront of treatments for inflammatory conditions (1). TNF signaling is mediated by binding to two cell-surface receptors: TNF receptor type 1 (TNFR1 or p55), expressed in most tissues, or TNF receptor type 2 (TNFR2 or p75), which is inducible and typically found in cells of the immune system (2). TNFR1 induces pro-inflammatory cascades and apoptosis, while TNFR2 has a role in cell survival, proliferation, and immune regulation (3, 4).

The structure of the related lymphotoxin α (LTα, previously called TNFβ) in complex with TNFR1 has shown that the trimeric cytokine binds three receptor molecules in a symmetrical way (5). The cytokine recruits two or possibly three TNFR1 molecules leading to clustering of the receptors, which results in downstream signaling. TNF has a similar structure as LTα and both compete for binding to the same receptors. Therefore, it was concluded that the structural insights provided by the analysis of the complex of LTα with p55 should also apply for TNF.

Inhibition of the TNF has been achieved by the anti-TNF biologic etanercept, antibodies like infliximab and adalimumab, or with the antibody fragment certolizumab, used to treat autoimmune diseases. However, these therapies have many adverse effects, and a lot of patients do not respond or poorly respond to initial treatment, or lose their response with maintenance therapy due to immunogenicity or other causes (6, 7). An alternative to total TNF blockade is to use a selective TNFR1 inhibitor to target pathogenic TNF signaling in autoimmune disease conditions such as RA. The TNF/TNFR2 pathway and its beneficial immunomodulatory signals and tissue homeostatic functions are thus maintained (8).

While the antibody structure usually comprises a heavy chain combined with a light chain, camelids have antibodies that only consists of a heavy chain (HCAbs) (9). Variable heavy (VH) chain domains of heavy-chain antibody (VHH) or Nanobodies™ are small antigen-binding fragments derived from HCAbs (4). They have advantages over conventional antibodies in that they are small (15 kDa) and robust, with low immunogenicity, a unique binding capability, and high solubility and stability (10). They are encoded by a single gene, requiring no posttranslational modifications and can be produced at high yields in bacteria and yeasts (11).

We generated and isolated Nanobodies™ that were screened for inhibition of the interaction between TNF and the TNFR2. The TNF-specific Nanobodies™ were shown to inhibit the interaction between the cytokine and its receptor in enzyme-linked immunosorbent assay (ELISA). In addition, it is shown that these Nanobodies™ inhibit the TNF-induced necrosis effect in a cell-based assay. These bioassay data suggest that TNF:TNFR1 cascade is also inhibited, which is in accordance with previously published structural analysis of interaction between LTα and TNFR1 (5). Their potency is better than for the commercially available TNF inhibitors, such as etanercept (Enbrel^®^), adalimumab (Humira^®^), and infliximab (Remicade^®^). However, these TNF antagonistic therapeutics act as bivalent molecules, resulting in higher avidity (12). Therefore, bivalent Nanobodies™ with either a short 9 amino acid GlySer linker or a longer 12 and 30 amino acid GlySer linker were constructed and characterized.

The X-ray structures of three complexes of these Nanobodies™ and TNF make it possible to rationalize the impact of linker length on the potency of these TNF binders. This knowledge supports the rational design of the most optimal bivalent Nanobody™ constructs that demonstrate efficacy in the TNF transgenic mouse model of spontaneous arthritis.

## Materials and Methods

### Production and Selection of Nanobodies™ Blocking TNF

The study was approved by the Ethical Committee of the Faculty of Veterinary Medicine, University Ghent, Belgium. Human TNF was produced in house (Ablynx NV, Belgium) as a recombinant protein in *Escherichia coli* using the method described by Marmenout et al. (13). Purification was according to the procedures described by Curnis and Corti (14).

Two llamas were immunized with TNF according to current animal welfare regulations, using the adjuvant Stimune (CEDI Diagnostics, Lelystad, The Netherlands). Two blood samples were collected from each animal as the source of B-cells. Total RNA was isolated according to the procedure described by Chomczynski and Sacchi (15). Random primed complementary DNA was prepared on total RNA, purified and subsequently used as template to amplify the Nanobody™ repertoire. The procedure to amplify and clone the Nanobody™ repertoire was based on a method described in Ref. (16).

For the selection of Nanobodies™ against TNF, a Nunc Maxisorp^®^ 96-well plate was coated with neutravidin and blocked, and biotinylated TNF was added to the wells. Phages were prepared as described by Marks et al. (17) and allowed to bind to the wells for 2 h at room temperature. Phages were removed, and the wells were washed 20 times with phosphate-buffered saline (PBS)/0.1% tween; elution of bound phage was done with 10 µM etanercept (Enbrel^®^) for 30 min at room temperature, or by denaturation with acid (0.2 M glycine pH 2.5) for 20 min at room temperature. Two rounds of selections were performed.

The ability of the Nanobodies™ to inhibit receptor–ligand interaction was analysed in ELISA. A 96-well Maxisorp plate was coated overnight at 4°C with 2 µg/ml etanercept in PBS. Plates were blocked with 1% casein solution (in PBS) for 2 h at room temperature. Nanobody™ samples were preincubated for 30 min at room temperature with biotinylated TNF (200 pM). The mixtures were added to the plates and incubated for 1 h at room temperature. Biotinylated TNF was detected using Extravidin alkaline phosphatase (Sigma; 1/2,000 diluted) and pNPP (Sigma; 2 mg/ml) as substrate.

### Formatting, Expression, and Purification of Nanobodies™

For construction of bivalent anti-TNF Nanobodies™, two separate PCR reactions were used to amplify the N-terminal and the C-terminal Nanobody™ subunits using oligo combinations containing sequences encoding a 9GS [(Gly)_4_Ser(Gly)_3_Ser], 12GS [(Gly)_3_(Ser)]_3_, and 30GS [(Gly)_4_(Ser)]_6_ linker to connect the different Nanobodies™. The N-terminal VHH PCR fragment was digested with SfiI and BamHI, and the C-terminal VHH PCR fragment was digested with BamHI and BstEII. Ligations and transformations were carried out as described earlier.

For the generation of bispecific Nanobodies™ consisting of two anti-TNF Nanobodies™ combined with one anti-albumin Nanobody™, three PCR reactions were performed for the amplification of the N-terminal, the middle, and the C-terminal Nanobody™ with oligonucleotide primers encoding the 9, 12, or 30 × Gly–Ser linker. The N-terminal VHH encoding PCR fragment was digested with SfiI and BamHI, the middle Nanobody™ fragment was digested with BamHI and BspEI, and the C-terminal VHH PCR fragment was digested with BspEI and BstEII.

Single *E. coli* clones were picked and grown in Luria Broth containing the appropriate antibiotics, and expression was induced with 1 mM isopropyl β-d-1-thiogalactopyranoside. Periplasmic extraction and immobilized metal affinity chromatography purification of the VHH proteins were performed according to Ref. (18). The VHH proteins were further purified by cation exchange and/or gel filtration and dialyzed into PBS.

### Affinity Measurements

Binding of Nanobodies™ to TNF was characterized by surface plasmon resonance in a Biacore 3000 instrument (Biacore International AB, Uppsala, Sweden). In brief, TNF was covalently bound to a CM5 sensor chip surface *via* amine coupling until an increase of 250 response units was reached. Remaining reactive groups were inactivated. Nanobody™ binding was assessed, and *K*_D_ values were calculated using the instruments software.

### Neutralizing Potency Measured in Cell-Based Assay

The TNF sensitive mouse fibroblast cell line L929s was utilized for measuring the anti-TNF activity of the selected Nanobodies™. L929 cells were grown until nearly confluent, plated out in 96-well microtiter plates at 5,000 cells per well, and incubated overnight. Actinomycin D was added to the cells at a final concentration of 1 µg/ml. Serial dilutions of the Nanobodies™ to be tested were mixed with a cytotoxic concentration of TNF (10 pM). After incubation for 30 min at 37°C, this mixture was added to the plated cells and incubated for 24 h at 37°C. Cell viability was determined by using the tetrazolium salt WST-1. Dose–response curves and IC_50_ values (potency) were calculated with GraphPad Prism. The mean potencies for individual Nanobody™ constructs and benchmark anti-TNF biologics were calculated from a number of independent bioassays as well as the SD.

### Size Exclusion Chromatography (SEC) of Complexes of TNF with Nanobodies™

Size exclusion chromatography of complexes of TNF with the different formats of VHH using a Superdex 200 HR 10/30 column was carried out according to the procedure described by Santora et al. (19). 20 µg (0.4 nmol) of human TNF in a volume of 100 µl (in PBS) was injected on the column. For analysis of Nanobody™–cytokine complexes, 20 µg (1.3 nmol) of monovalent antibody fragment was mixed with 20 µg (0.4 nmol) of cytokine in 100 µl volume and after 30 min preincubation at room temperature loaded on the column. For the bivalent nanobody construct, 20 µg (0.7 nmol) antibody fragment in 100 µl volume was applied on the column. SEC was performed with the mixture of 20 µg (0.4 nmol) of cytokine and 40 µg (1.3 nmol) of bivalent nanobody in a volume of 100 µl, which was preincubated for 30 min at room temperature. The column has been calibrated one month before the analysis of the bivalent constructs with the Gel Filtration Standards [BioRad, catalog number 151-1901 containing bovine thyroglobulin (MW 670 kD), bovine γ-globulin (158 kD), chicken ovalbumin (44 kD), horse myoglobin (17 kD), and vitamin B12 (1.35 kD)]. This procedure enabled determination of the molecular mass of the complexes and, hence, their stoichiometry.

### X-ray Structures Determination

The complex between TNF and VHH#1 was crystallized by mixing 100–300 nl of purified complex (8 mg/ml in HEPES 10 mM pH 7.0) with 100 nl of precipitant solution (20% PEG3000, 0.2 M NaCl, and 0.1 M HEPES at pH 7.5) and equilibrating 100 µl of precipitant solution. A crystal was exposed at beamline ID14-1 (ESRF, Grenoble, France), and a complete dataset was collected at 2.15 Å resolution (Table 1). Data were integrated with XDS and scaled using Xscale (20) (Table 1). The structure was solved by molecular replacement with Molrep (21) using the TNF trimer from Protein Data Bank (PDB) entry 1TNF and the framework region (FR) of the VHH domain from PDB entry 1HCG as search models. Refinement was carried out with cycles of autoBUSTER (22) alternated with manual rebuilding with Coot (23).

**Table 1:** Data collection and refinement statistics.

Data collection	TNF–VHH#1	TNF–VHH#2	TNF–VHH#3
Protein Data Base	5m2i	5m2j	5m2m
Source	ESRF ID14-1	ESRF ID14-1	ESRF ID14-3
Space group	P2_1_2_1_2_1_	P6_3_	C2
Cell (Å), angle (°)	*a* = 110.3, *b* = 117.4, *c* = 141.9	*a* = *b* = 87.3, *c* = 62.7	*a* = 145.4, *b* = 83.8, *c* = 150.1, β = 128.8
No. monomers in the AU	6	1	6
Resolution limits (Å)	50–2.15 (2.2–2.15)	50–1.9 (1.95–1.9)	30.0–2.3 (2.42–2.3)
*R*_merge_	0.127 (1.11)	0.035 (0.10)	0.09 (0.32)
CC1/2	0.997 (0.72)	0.999 (0.98)	0.999 (0.96)
Unique reflections	100,770 (7,354)	21,089 (1,499)	62,613 (9,101)
Mean [(I)/SD(I)]	8.8 (1.5)	23.5 (11)	11.2 (4.1)
Completeness (%)	99.5 (95.6)	97.9 (94.6)	99.9 (99.9)
Multiplicity	4.15 (4.0)	2.9 (2.7)	4.1 (4.0)
**Refinement**			
Resolution (Å)	46.5–2.15 (2.21–2.15)	48.3–1.9 (2.0–1.9)	30.0–2.3 (2.36–2.3)
Number of reflections	100,502 (2,376)	21,089 (2,723)	62,613 (4,594)
Number of protein/water atoms	12,521/747	1,966/351	12,810/711
Test set reflections	5,026 (369)	1,045	2,988 (231)
*R*_work/_*R*_free_	0.208/0.238 (0.234/0.258)	0.16/0.196 (0.16/20.0)	0.211/0.248 (0.212/0.244)
RMSD bonds (Å)/angles (°)	0.008/1.17	0.010/1.11	0.008/1.11
B-Wilson/B-mean (Å)	36.5/44.2	15.6/19.8	35.1/44.5
Ramachandran: preferred/allowed/outliers (%)	96.4/3.1/0.5	97.6/2.4/0	95.8/3.7/0.5

*Numbers between brackets refer to the highest resolution bin*.

Crystals of the complex between TNF and VHH#2 were obtained by mixing 100–300 nl of purified complex (11 mg/ml in HEPES 10 mM pH 7.0) with 100 nl of precipitant solution (12% PEG4000, 130 mM NaCl, 366 mM CaCl_2_, 70 mM CAPS pH 9.0, and 30 mM MES pH 8.0) and equilibrating against 100 µl of precipitant solution. Data to 1.9 Å resolution were collected from a single crystal at beamline ID14-1 (ESRF, Grenoble, France) (Table 1). Data were integrated with XDS and scaled using XSCALE (20) (Table 1). The structure was determined by molecular replacement with Molrep (21) using a single TNF monomer and a single VHH domain stripped from its complementarity determining region (CDR) loops and taken from the TNF–VHH#1 complex as search models. Refinement was performed as described above.

Crystallization of the complex between TNF and VHH#3 was achieved by mixing 100–300 nl of protein (8–10 mg/ml in HEPES 10 mM pH 7.0) with 100 nl of precipitant solution (9% PEG3350, 8% PEG-MME550, 130 mM NaSO_4_, 70 mM BTP, 30 mM MES, and 3 mM ZnSO_4_, 7.0 < pH < 8.0) and equilibrating against 100 µl of precipitant solution. A crystal was exposed at beamline ID14-3 (ESRF, Grenoble, France), and a complete dataset was collected at 2.3 Å resolution (Table 1). Data were integrated with XDS and scaled using XSCALE (20). The structure was determined by molecular replacement using the coordinates of the TNF trimer and VHH#1 stripped from its CDR loops as search models. Refinement was performed as described above for the other two TNF complexes.

Protein contacts were analyzed using PISA (24). Figures were prepared with Pymol (Pymol, Schrödinger). Coordinates and structure factors have been deposited at the PDB as entries 5m2i, 5m2j, and 5m2m for the complexes with VHH#1, VHH#2, and VHH#3, respectively.

### Arthritis Treatment in the Tg197 Mouse Model

The transgenic Tg197 model was used to investigate the potency of the different VHH (VHH#1 and VHH#3) antibodies, as described in Keffer et al. (25). Briefly, Tg197 mice carry a human TNF transgene, with its 3′-untranslated region replaced by a sequence from the 3′-untranslated region of the beta-globin gene, thereby allowing deregulated human TNF gene expression. By 4 weeks of age, all human TNF expressing Tg197 mice spontaneously develop a severe bilateral, symmetric, erosive, and disabling polyarthritis similar to RA. Treatment of these arthritic mice with monoclonal antibodies (mAb) against human TNF can prevent development of the disease.

Using this model, 13 groups of 8 mice each were assigned to one of four treatment regimens: PBS treatment, VHH#1-based bivalent molecule, VHH#3-based bivalent molecule, or the biologic etanercept (Enbrel^®^; a TNFR-Fc fusion protein).

The VHH#3-based bivalent construct consisted of the following, in sequence: a VHH#3 molecule, a 9GS linker, a VHH#3 molecule, a 9GS linker, and finally, an antihuman serum albumin VHH (anti-HSA VHH) at the carboxy-terminus to avoid rapid clearance of the compound from circulation (i.e., a construct of: VHH#3-9GS-VHH#3-9GS-HSA VHH). The VHH#1-based bivalent construct consisted of the following, in sequence: VHH#1, a 9GS linker, the anti-HSA VHH, another 9GS linker, and a VHH#1 molecule (i.e., a construct of: VHH#1-9GS-HSA VHH- 9GS-VHH#1).

Doses of 1, 3, 10, and 30 mg/kg were administered intraperitoneally twice weekly, starting week 3 after birth. The arthritic scoring system (26) was applied based on the macroscopic changes observed in joint morphology on both ankle joints using the following scores: 0 = no arthritis (normal appearance and flexion); 0.5 = onset of arthritis (mild joint swelling); 1 = mild arthritis (joint distortion); 1.5 = as above, but with finger deformation, less strength on flexion; 2 = moderate arthritis (severe swelling, joint deformation, no strength on flexion); 2.5 = as above, but with finger deformation in paws; 3 = heavy arthritis (ankylosis detected on flexion and severely impaired movement).

Arthritic score (AS) was recorded weekly on both ankle joints, and average scores were calculated. Statistical significance was tested using analysis of variance for multiple groups. When significant differences were observed, pairwise testing was performed using Tukey’s multiple comparison test. ASs were statistically evaluated at the end of study, i.e., at 10 weeks of age.

## Results

### Identification and Potency of Antagonistic Anti-TNF VHH

For isolation of Nanobodies™ that act as antagonists of TNF, two llamas were immunized with human TNF, and phage display libraries were generated using RNA derived from peripheral blood lymphocytes. Selection was performed by competitive elution with an excess of Enbrel on biotinylated TNF, captured by immobilized streptavidin (27). The principle of competitive elution is based on saturating all receptor binding sites on the cytokine, thereby preventing rebinding of dissociated phage antibodies, and thus enrichment for antagonistic VHH. A similar approach was used by others for the isolation of human immunodeficiency virus-1 neutralizing VHH (28). Indeed, using this methodology on TNF led to the identification of only “blocking” (antagonistic) Nanobodies™, i.e., VHH#2 and VHH#3 from one llama and VHH#1 from the other llama.

These three Nanobodies™ each represent large families of affinity variants, which contain somatic mutations in the CDR and, to a lesser extent, in the FRs (16, 29). For VHH#3, even (lower affinity) family members exist with a deletion of two amino acids in CDR1, probably as the result of gene conversion during affinity maturation in the llama (30).

Recently, such variants of the anti-TNF VHH have also been identified *via* B-cell display methods [unpublished], which do not suffer from polymerase chain reaction artifacts during library construction. This confirms the occurrence of circulating B cells derived from an ancestor B-cell clone as a consequence of the *in vivo* maturation process.

The VHH encoding gene segments were recloned in an *E. coli* expression vector with or without the carboxy-terminal c-MYC and/or hexa-histidine tags. After expression and purification, VHH were tested in the bioassay for their neutralizing capacity. Murine L929 cells expressing the mouse receptor were used for testing the VHH in combination with the human cytokine. By sensitizing the cells with actinomycin D, picomolar amounts of TNF were sufficient to induce the cytotoxic effect, which was taken as the assay read-out. All three VHHs were found to have low nanomolar potencies in line with the measured affinities for TNF (Table 2).

**Table 2:** Potency (IC_50_) and affinity (*K*_D_) of monovalent and bivalent anti-TNF-Nanobody™ constructs.

Nanobody linker	IC_50_ mean (nM)	IC_50_-SD (nM)	*K*_D_ (nM)	Ratio IC_50_	Minimal linker length^a^	Nr measures
VHH#1	0.242	0.122	0.54	1		21
VHH#1-9GS-VHH#1	0.078	0.047		3.1	20	8
VHH#1-30GS-VHH#1	0.021	0.012		12		16
VHH#2	0.748	0.153	0.13	1		27
VHH#2-9GS-VHH#2	0.236	0.049		3.2	18	4
VHH#2-30GS-VHH#2	0.015	0.005		50		21
VHH#3	1.503	0.84	1.5	1		4
VHH#3-9GS-VHH#3	0.019	–		80	12	1
VHH#3-12GS-VHH#3	0.012	0.007		125		7
VHH#1-9GS-VHH#3	0.059	0.018		1	20	13
VHH#3-9GS-VHH#1	0.006	0.002		10	9	8
Etanercept	0.013	0.006		–		71
Adalimumab	0.127	0.058		–		67
Infliximab	0.144	0.061		–		68

*GS, amino acid glycine–serine linker; VHH, variable-domain heavy-chain region*.

*Etanercept (Enbrel^®^), adalimumab (Humira^®^), and infliximab (Remicade^®^)*.

*Ratio IC_50_ refers to ratio of IC_50_ of monomeric VHH and bivalent construct*.

*^a^Minimum possible linker length (number of amino acids) calculated from the 3D structure*.

### Format Engineering of Nanobodies™

Because of the trimeric nature of TNF, we investigated whether the avid binding of a mAb such as infliximab contributes to its potency. Indeed, a considerable difference in potency was observed when the Fab fragment prepared by proteolytic digestion of infliximab was tested in the bioassay and compared with the intact antibody. The IC_50_ of the monovalent Fab fragment was ~2 nM; compared with that of the bivalent immunoglobulin-G format (IC_50_ ~ 70 pM), the potency of the Fab was approximately 30-fold lower.

VHHs are strictly monomeric proteins and do not show any tendency to aggregate in multimers like single-chain variable fragments (scFv). Bivalent and bispecific formats were constructed by using linkers of variable length to fuse the VHHs. As a linker between the two VHHs, either a stretch of 9GS, 12GS, and 30GS sequence was used. Depending on the VHH and the length of the linker, a dramatic increase in potency was observed.

### Binding and Activity Determination

The affinity constants of binding to TNF for each of the three VHHs were determined by Surface Plasmon Resonance. The measured binding constants (*K*_D_) are 540 pM, 130 pM, and 1.5 nM for VHH#1, VHH#2, and VHH#3, respectively. In line with this, the IC_50_ values measured in the bioassay are in a similar range of between 240 pM to 1.5 nM (Table 2).

With regard to the bivalent VHHs, potencies increased when compared with the monovalent building blocks (Table 2). For the VHH#1-based bivalent constructs, measurements indicate a threefold increase in potency with a short 9GS linker and a greater increase in potency, by a factor of 12, with a longer 30GS linker. For VHH#2-based bivalent constructs, a threefold increase in potency is also observed with the 9GS linker, while a 50-fold increase in potency was measured with a 30GS linker. In contrast, for VHH#3-based bivalent constructs, even a short linker of 9GS allows an increase in potency by a factor of 80, while an increase of 120-fold is observed when using the 12GS linker (potency of 12 pM). However, such a low picomolar potency is not achieved with an Fc fusion, where the VHH is linked directly to the hinge region and to the constant CH2/CH3 domains of human IgG1 (i.e., the human version of the heavy-chain antibody format). The Fc derivative from VHH#3 has a potency of ~100 pM as compared to 1.5 nM for the monovalent VHH#3, showing that avidity-mediated binding does improve its efficacy, but not to the degree of that seen with the bivalent constructs (data not shown).

Increases in potency were also obtained when the two different Nanobodies™ VHH#1 and VHH#3 were linked with the short 9GS linker to create bispecific constructs, either with VHH#1 first or with VHH#3 first (Table 2). When VHH#3 is placed first, a 10-fold greater potency is obtained than when VHH#1 is first (Table 2). The potency of VHH#3-9GS-VHH#1 is 6 pM and is therefore at least twofold better than the potency of etanercept. Indeed, 10 pM was the amount of TNF used in the bioassay, thus representing the limit of sensitivity. This indicates that the position of the VHH plays an important role in binding, as previously observed (31), and may be determined by the exact epitope to which the respective Nanobodies™ bind.

### Intramolecular Binding

**Figure 1:**
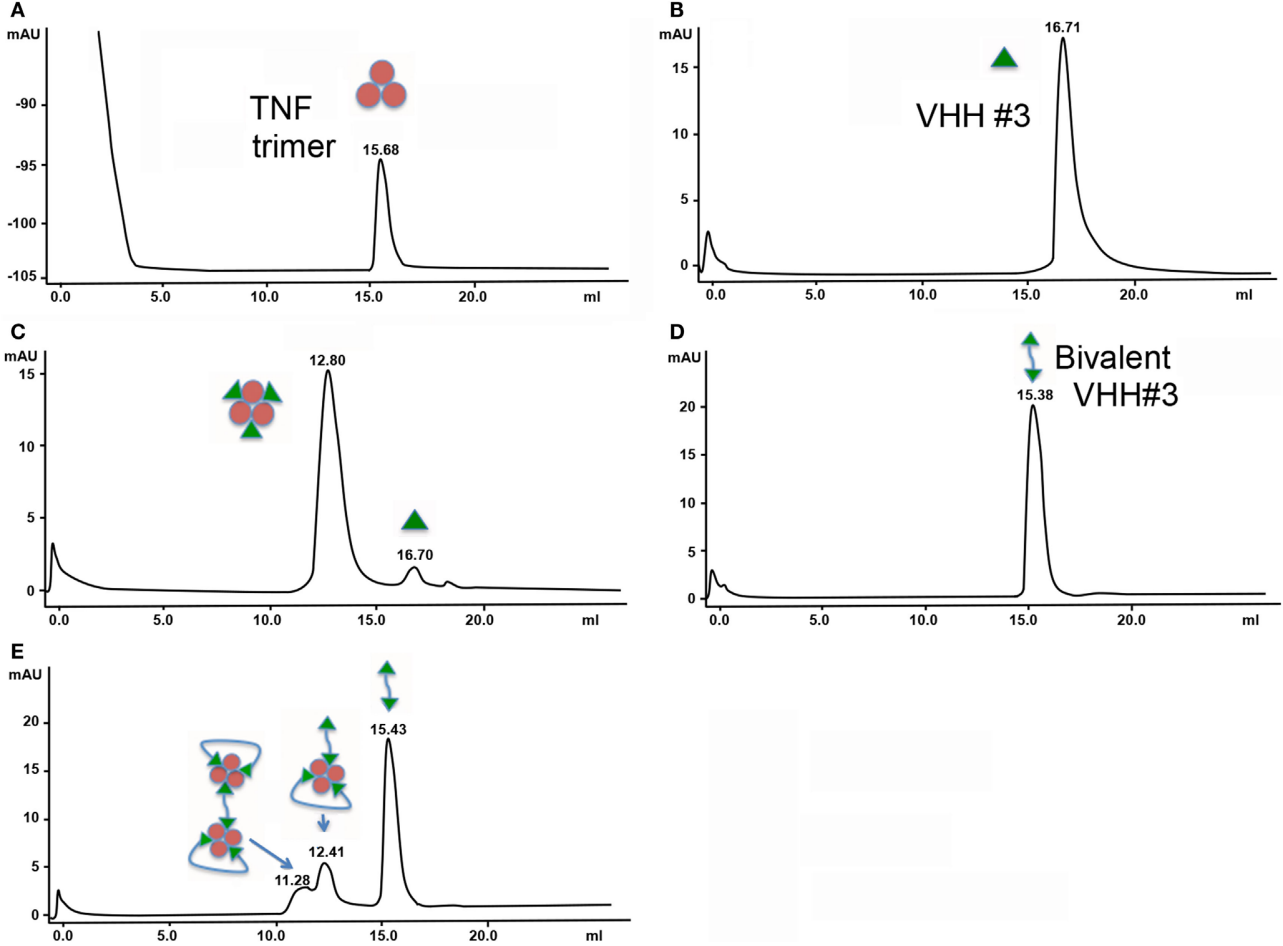
Size exclusion chromatography of complexes of tumor necrosis factor (TNF) with the different formats of VHH. **(A)** 0.4 nmol TNF. **(B)** 1.3 nmol VHH#3. **(C)** 0.4 nmol TNF + 1.3 nmol VHH#3. **(D)** 0.7 nmol bivalent VHH#3 (VHH#3-12GS-VHH#3). **(E)** 0.4 nmol TNF + 1.3 nmol bivalent VHH#3 (VHH#3-12GS-VHH#3).

The molecular masses and hence stoichiometries for the different TNF complexes (monovalent and bivalent VHH constructs) were determined by SEC. The trimeric TNF and the monovalent VHH molecules appear at elution times of 16.71 min (MW of around 50 kDa) and 15.68 min (MW 22.5 kDa), respectively (Figures 1A,B). By mixing 0.4 nmol of TNF with 1.3 nmol of VHH#3, a major peak and a smaller peak eluted with retention times of 12.8 and 16.7 min, respectively (Figure 1C). The major peak, which according to the elution volume has a molecular weight of around 210 kDa (elutes a bit earlier than the 158 kDa standard), is attributed to a TNF/VHH#3 complex with a 1:3 stoichiometry (three Nanobodies™ on one TNF trimer), and the smaller peak to the VHH#3 molar excess [1.3 nmol − (3 nmol × 0.4 nmol)].

Similar experiments were performed with the bivalent VHH#3 construct containing the 12GS linker. This bivalent VHH#3 construct appears at a retention time of 15.38 min (MW of around 45 kDa; Figure 1D). We then mixed 0.4 nmol of TNF with 1.3 nmol bivalent VHH#3. This mixture corresponds to a small molar excess if only one of the two Nanobodies™ binds to TNF [1.3 − (0.4 × 3) = 0.1 nmol], while it corresponds to a large molar excess if the two Nanobodies™ bind to TNF [1.3 − (0.4 × 2) = 0.5 nmol] as is shown in Figure 1E. After injection of this mixture, three peaks were observed: a major peak at 15.43 min, corresponding to the free bivalent VHH#3 (about 0.6 nmol), and two smaller peaks at lower retention times of 12.41 and 11.28 (Figure 1E). The amount of free bivalent VHH#3 is above the value of 0.5 nmol calculated above for the situation the two bivalent nanobodies can bind to a single TNF molecule. With regard to the latter peaks, both are at lower retention times compared to the TNF trimer/3× VHH#3 complex, indicating that the complexes are of larger molecular weight. The larger peak of the doublet, at 12.41 min, elutes just a bit earlier than the complex of TNF with three monomeric Nanobodies™ (12.80 min; indicates MW of around 240 kDa) meaning that it must contain more mass than three Nanobody™ subunits. This could implicate that the complex consists of one TNF molecule with two bivalent Nanobody molecules as depicted in Figure 1E, in which one bivalent molecule binds to two receptor interaction sites, whereas the other bivalent molecule binds with one arm to a single receptor interaction site. The peak at 11.28 min has an even larger molecular weight; it elutes just after the dimeric thyroglobulin peak of 330 kDa. The difference between the two peaks is around 80 kDa, which could account for one additional TNF and one bivalent VHH molecule. This may account for a complex in which four bivalent VHH molecules bind to two TNF trimers, hence its larger molecular weight.

### Structures of the Complexes

**Figure 2:**
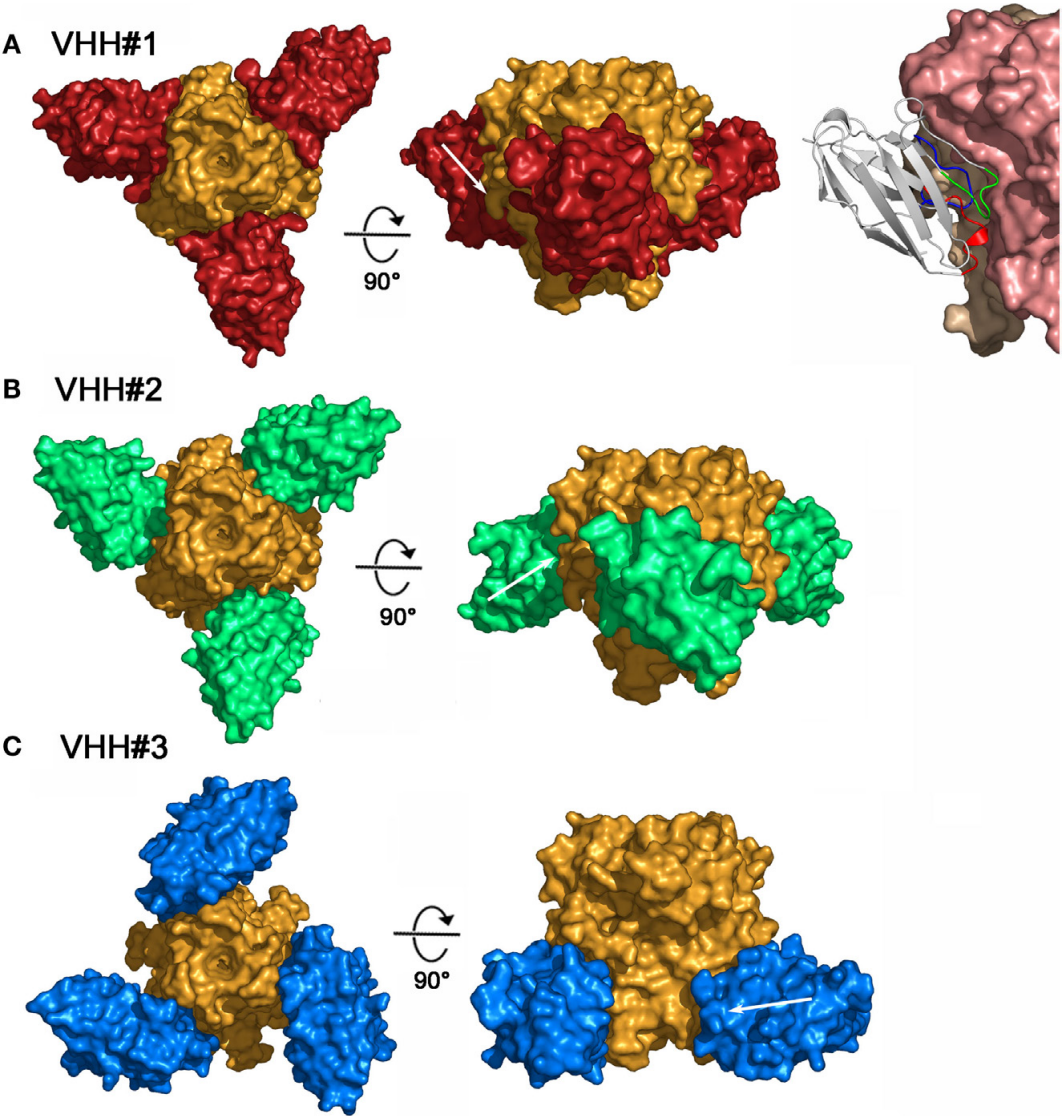
Three-dimensional structures of the three tumor necrosis factor (TNF)–Nanobody™ complexes. **(A)** Complex TNF–VHH#1; TNF and VHH#1 surfaces are colored orange and red, respectively. Shown right: details of the interaction of VHH#1 with TNF; VHH#1 in ribbon representation. **(B)** Complex TNF–VHH#2; TNF and VHH#2 surfaces are colored orange and green, respectively. **(C)** Complex TNF–VHH#3; TNF and VHH#3 surfaces are colored orange and blue, respectively.

**Figure 3:**
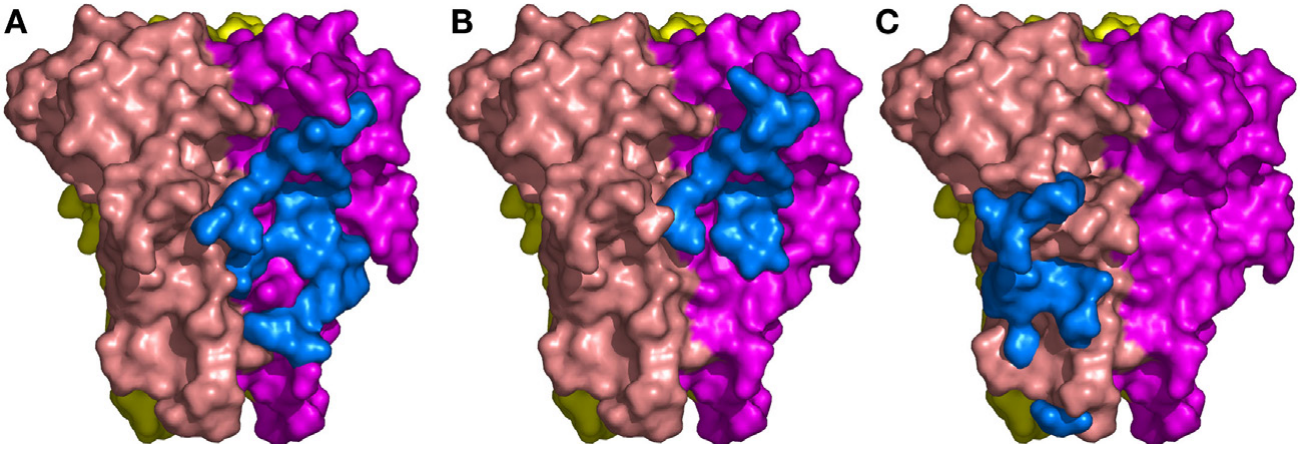
The binding sites of the three Nanobodies™ on tumor necrosis factor (TNF) trimer. **(A)** Complex TNF–VHH#1. **(B)** Complex TNF–VHH#2. **(C)** Complex TNF–VHH#3. TNF-monomer surfaces are colored orange, violet, and yellow. The residues of the Nanobodies™ in interaction with TNF are displayed as blue surfaces.

To better understand and rationalize the activities of the three Nanobodies™, the crystal structures of the complexes of VHH#1, VHH#2, and VHH#3 with TNF were determined. In agreement with SEC data, each of the three Nanobodies™ associates with the trimeric TNF to form a hetero-hexameric TNF-Nanobody™ complex (Figure 2). VHH#1 attaches to the concave surface of the outer β-sheet of TNF and contacts two TNF monomers simultaneously (Figures 2 and 3). VHH#1 covers 1,246 Å^2^ of the TNF solvent-accessible surface area, of which 902 Å^2^ belongs to one TNF monomer and 274 Å^2^ to the other monomer (Table 3). Its three CDRs interact with TNF, but CDR2 and especially CDR3 are predominant in the interaction, as commonly observed in other Nanobody™ complexes (Table 3). VHH#1 is aligned almost parallel to the TNF surface (when comparing the orientations of their β-strands), and its CDR2 lines the TNF surface (Figures 2 and 3).

**Table 3:** Water accessible surface area (in Å^2^) of TNF or VHH covered when complexed.

Part	VHH#1	VHH#2	VHH#3
VHH	1,246	745	736
TNF trimer	1,176	746	749
TNF-monomer 1	902	571	749
TNF-monomer 2	274	175	–
CDR1	236	272	73
CDR2	409	322	314
CDR3	599	131	123

*CDR, complementarity determining region; TNF, tumor necrosis factor; VHH, variable-domain heavy-chain region*.

The binding area of VHH#2 coincides almost totally with that of VHH#1 (Figure 3; Table 4) despite significant differences in CDR lengths and conformations, and a different orientation of both VHH domains relative to the antigen. VHH#2 covers 745 Å^2^ of the TNF solvent-accessible surface area, 571 Å^2^ on one TNF monomer and 175 Å^2^ on the other monomer (Table 3). However, the orientation of VHH#2 is more perpendicular to TNF compared to that of VHH#1, which results in a smaller surface area of interaction (Figures 2 and 3; Table 3). VHH#2 is unusual in that most of the contacts with the antigen involve CDRs 1 and 2. CDR3, which normally dominates Nanobody™-antigen interactions, contributes least to the contact surface (Table 3). The CDR3 of VHH#2 is among the shortest observed for Nanobodies™ and the FR after the CDR3 sequence deviates from the normal framework-4 structure: the C-terminal β-strand is shortened in order for the CDR3 loop to connect with the previous β-strand.

**Table 4:** TNF residues in contact (*d* < 3.8 Å) with the three VHHs.

TNF	Residues	VHH#1	VHH#2	VHH#3
20	B			
21	B			X
22	B			X
23	B			X
24	B			X
25	B			X
65	B			X
66	B			X
67	B			X
70	B			X
72	B			
73	B			
74	B			
77	B			
79	B			
81	B			
83	B			
83	B			
88	B			
89	B			
90	B			
91	B			
92	B			
97	B			
107	B			
135	B			
136	B			
137	B			
138	B			
139	B			
140	B			
141	B			
115	C			
145	C			
146	C			
147	C			

*Shaded boxes represent residues within TNF that are in contact with residues of the indicated Nanobody*.

*TNF, tumor necrosis factor; VHH, variable-domain heavy-chain region*.

In contrast, VHH#3 binds to a different epitope that does not overlap with the epitopes of VHH#1 and VHH#2, and that is located toward the thinner end of the trimer, “below” the VHH#1 epitope (Figures 2 and 3; Table 4). Each VHH#3 molecule contacts only a single TNF monomer and covers 736 Å^2^ of accessible surface area of TNF (Table 3). Again, all three CDR loops contribute to antigen binding, with CDR2 being dominant and CDR1 showing the smallest contribution despite its relatively long length (Table 3).

### TNF Receptor Neutralization

The 3D structure of TNF in complex with TNFR is not known. However, given that TNF and LTα bind equally well to TNFR1 (p55) and TNFR2 (p75) and considering the high structural identity between TNF and LTα, a plausible model for the TNF/TNFR1 complex can be obtained by superimposing TNF onto LTα in its complex with the ectodomain of the TNFR1 p55 receptor (PDB entry 1TNR) (5). In this model, each TNFR1 p55 monomer covers about 1,200 Å^2^ of one of the three TNF monomers without significant steric clashes.

**Figure 4:**
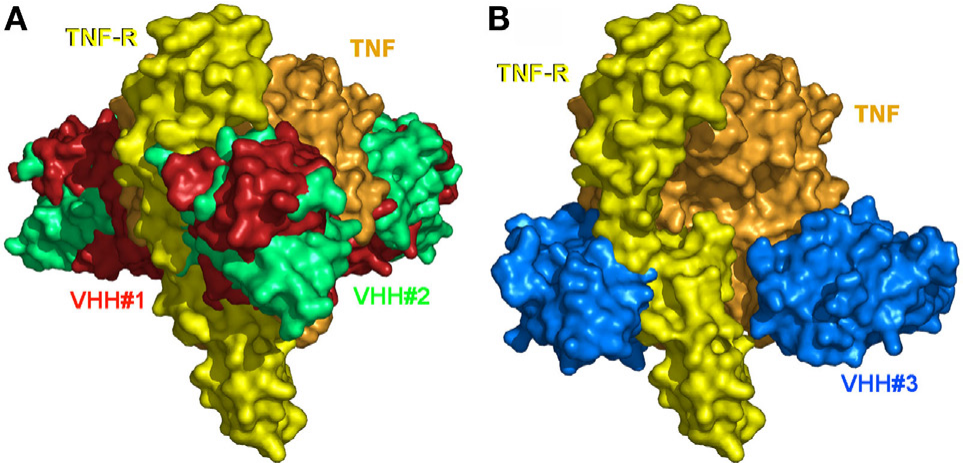
The binding sites of the three Nanobodies™ and of the extracellular domain of the tumor necrosis factor (TNF) receptor p55 (TNFR1) mapped on TNF. **(A)** VHH#1, VHH#2, and TNFR1. **(B)** VHH#3 and TNFR1. The TNF trimer surface is colored orange. The TNFR1 surface of a unique monomer is colored yellow. Surfaces of VHH#1, VHH#2, and VHH#3 are colored red, green, and blue, respectively. Note the superposition of the Nanobodies’ surfaces with those of TNFR1.

The structures of the TNF/VHH complexes reported here were superimposed onto that of the TNF/TNFR complex. The binding areas of VHH#1, VHH#2, and VHH#3 overlap in part with that of TNFR (Figure 4). VHH#1 and VHH#2 occupy the central area of the receptor binding site, while VHH#3 occupies the “bottom” of the receptor binding area (Figure 4). Furthermore, whereas two molecules of VHH#1 or VHH#2 interact with two TNF subunits on either side, only one molecule of VHH#3 interacts with a single TNF subunit (Figure 3). However, considering the dramatic steric overlap between TNFR and all three VHH molecules, it is clear that the three VHH molecules can inhibit the interaction between TNF and TNFR.

Notably, the interaction areas of VHH#1 and VHH#3 are quite distinct, and they can bind simultaneously to TNF at a single receptor binding site, ensuring a possible biparatopic binding. This observation explains why the VHH#1-9GS-VHH#3 bispecific construct yields the highest potency ever observed for any TNF antagonist (6 pM; Table 2).

### Arthritis Treatment in the Tg197 Mouse Model

Bivalent constructs of VHH#1 and VHH#3, which recognize completely different epitopes on the cytokine, were tested in the Tg197 transgenic human TNF model for polyarthritis, in which mice constitutively produce the human cytokine and develop acute arthritis. VHH#2 was not included in this experiment, because it recognizes a similar epitope in the bulky part of the cytokine as VHH#1 does, but as a monovalent nanobody with a lower affinity and potency than VHH#2. Thirteen groups comprising eight mice each were assigned to one of four treatment regimens: PBS control, VHH#1-based bivalent molecule, VHH#3-based bivalent molecule, or etanercept.

The bivalent constructs consisted of two molecules of VHH#1 or VHH#3, and one molecule of an anti-HSA VHH to avoid rapid clearance of the compound from circulation. For VHH#1 a bispecific construct was generated with the anti-albumin Nanobody™ (human–mouse cross-reactive) in the middle position fused with the 9 amino acid GS linker to both VHH#1 Nanobodies™, thereby bridging a distance of approximately 30 amino acids between the two TNF Nanobodies™, which according to the structural studies should be sufficient for enforcing intramolecular binding to the cytokine. For VHH#2 the albumin binding Nanobody™ was placed in the carboxy-terminal position thereby supporting the use of the smaller 9GS linker for directly connecting the two VHH#2 units that would be required for intramolecular binding. The potencies of the bivalent VHH#1 Nanobody™ (VHH#1-9GS-HSA VHH- 9GS-VHH#1) and the bivalent VHH#3 Nanobody™ (VHH#3-9GS-VHH#3-9GS-HSA VHH) in the cell-based potency assay were 22 and 18 pM, respectively. The potency of etanercept in this assay was 14 pM (data not shown). The amount of TNF used in the bioassay was 10 pM, which therefore represents the limit of sensitivity.

**Figure 5:**
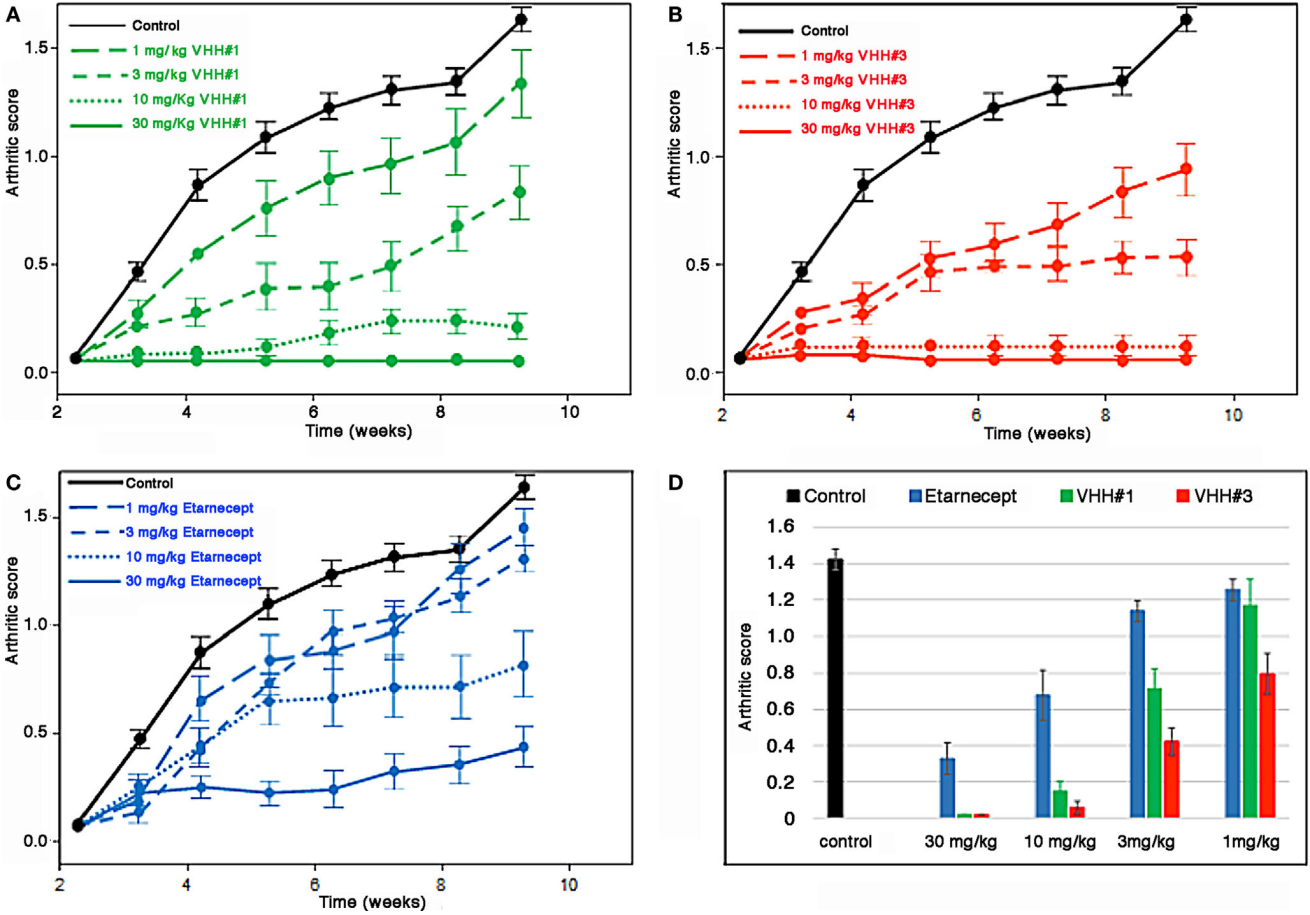
Efficacy of Nanobodies™ in Tg197 mouse model. Human tumor necrosis factor (TNF) transgenic mice were treated at week 3 after birth (one week before arthritic symptoms develop, i.e., in a prophylactic setting) with bivalent anti-TNF/anti-albumin Nanobody™ constructs or etanercept biweekly. Arthritic scores were recorded weekly up to week 10. **(A)** Bivalent VHH#1 (VHH#1-9GS-HSA VHH-9GS-VHH#1) construct versus phosphate-buffered saline (PBS) control. **(B)** Bivalent VHH#3 (VHH#3-9GS-VHH#3-9GS-HSA VHH) construct versus PBS control. **(C)** Etanercept versus PBS control. **(D)** Results for all groups at week 10.

The results of this study show that bivalent Nanobodies™ suppressed the development of arthritis in a dose-dependent fashion. In particular, administration either of bivalent VHH#3 at four different doses (1, 3, 10, or 30 mg/kg) or bivalent VHH#1 at three different doses (3, 10, or 30 mg/kg) had a significant effect (*p* < 0.05) in the amelioration of clinical scores (Figure 5) in comparison to the PBS treated group. In contrast, administration of VHH#1 at 1 mg/kg did not show statistically significant differences in clinical scores in comparison to the vehicle treated group (*p* > 0.05). Amelioration of arthritis by treatment of mice with etanercept at 10 or 30 mg/kg was also statistically significant in comparison to PBS treated group, whereas at lower doses (1 or 3 mg/kg) treatments with etanercept were no longer statistically significant. Complete attenuation of disease development was observed mice treated with 30 or 10 mg/kg of bivalent VHH#3, and in animals treated with 10 and 30 mg/kg of bivalent VHH#1. These results show that bivalent VHH#3 is improving clinical scores in all four doses tested ranging from 1 to 30 mg/kg. Bivalent VHH#3 is more potent than bivalent VHH#1 construct or etanercept. Bivalent VHH#1 improves the clinical scores at doses ranging from 3 to 30 mg/kg and is comparable to etanercept in terms of efficacy.

## Discussion

The action of TNF can be inhibited by antibodies or trap molecules targeting the cytokine (32). Herein, llama VHH domains were generated for the engineering of bivalent constructs, which antagonize the binding of TNF to its receptor with low picomolar potencies. Three monomeric VHHs, VHH#1, VHH#2, and VHH#3 were identified, which bind TNF with sub-nanomolar affinities. We previously observed that engineering VHH into bivalent constructs improved their potency, as demonstrated for other targets including mouse TNF (33), CXCR4 (34), and viruses (35). Anti-TNF VHH fused to a scFv or another VHH that recognized cell-surface markers of myeloid cells could capture TNF produced by these cells thereby restricting the bioavailability of the cytokine and hence such bispecific constructs were able to prevent the pathogenic effect in an *in vivo* model (36).

VHH#1 and VHH#2 bind to largely the same epitope at the center of the interaction area of TNF with TNFR (Table 4). The latter docks into a groove formed by the interface of two TNF monomers in the TNF trimer. Also, the CDR loops of VHH#1 and VHH#2 dock into this groove, although the specifics of the interactions are quite different due to a different orientation of the VHH module relative to the TNF structure. The recognition of clefts and grooves is a quite common feature of Nanobodies™ (37) and is due to the small size of the Nanobody™ relative to a scFv. Indeed, when both Nanobodies™ would be replaced by classical VH domains in the context of a scFv, large steric overlap would be present between the TNF trimer and the variable light (VL) domain, preventing the same mode of binding.

VHH#3 binds to a different epitope, and its recognition site on TNF only marginally overlaps with those of TNFR or VHH#1. There is no overlap between the recognition surfaces of VHH#2 and VHH#3, but steric overlap between the bodies of these two VHHs is still significant and should prevent coincident binding. This explains why VHH#3 was also identified as a blocking antibody.

Bivalent and bispecific Nanobodies™ created as VHH tandems show enhanced affinities and potencies that are dependent of the length of the linker used (Table 2). The structures of the three VHH complexes made it possible to rationalize these findings. The shortest distances between the N-terminus of one VHH and the C-terminus of another VHH in the complexes involving VHH#1 and VHH#2 are 69 and 67 Å, respectively. Taking into account the orientations of these termini relative to each other and that a linker needs to avoid steric clashes with the TNF antigen, a minimum linker length of at least 20 amino acids is expected to be required for the VHH#1 bivalent construct, and 18 amino acids for the VHH#2 bivalent construct. This explains the 12- and 50-fold enhancements in affinity for VHH#1 and VHH#2, respectively, when using the 30 amino acids GlySer linker. Indeed, the observed smaller molecular species using SEC argues for the recognition of two binding sites on the same TNF trimer by these bivalent Nanobody™ constructs.

For the constructs with a short nine amino acids GlySer linker, simultaneous binding of both VHH#1 or VHH#2 modules to the same TNF trimer is not possible. The moderate threefold increase in affinity when employing the short nine amino acids GlySer linker is likely due to a rebinding effect similar to what is observed for galectins binding to asialofetuin (38) or for the recognition of phosporylation sites on Sic1 by Cdc4 (39).

The shortest distance between the N- and C-termini of two VHH#3 molecules bound simultaneously to the TNF trimer is 51 Å, suggesting a minimal required linker length of only 12 amino acids. This is in agreement with 125-fold increase in potency for the bivalent construct with a 12 amino acids linker. Interestingly, the construct with a shorter nine amino acids linker shows an 80-fold increase in potency, indicating that the N- and/or C-terminus of VHH#3 has sufficient conformational flexibility to accommodate such a short linker while still bridging both binding sites.

We determined the three-dimensional structures of the three VHHs in complex with TNF after bispecific constructs had been generated with the two Nanobodies™ VHH#1 and VHH#3 linked *via* a nine amino acid GlySer linker, either with VHH#3 first or with VHH#1 first (Table 2). Data show that the combination with VHH#3 at the N-terminal position is superior in potency to the construct with VHH#1 at that position by an order of magnitude (Table 2). When superimposing the structures of TNF–VHH#1 and TNF–VHH#3, it becomes clear that only one of the two different combinations of these Nanobodies™ linked to each other with the short linker in a bispecific construct should be able to bind in an intramolecular fashion, since the second leads to steric clashes. The bispecific Nanobody™ VHH#3-9GS-VHH#1 can bind in an intramolecular fashion to the TNF-monomer subunit, whereas the bispecific construct with VHH#1 at the N-terminal position required a linker of at least 20 amino acids in order to achieve intramolecular binding.

Superiority of bivalent over monomeric constructs was recognized many decades ago, when such improvement was termed “avidity” (40). Superiority was due to the localization of the second binder close to the second binding site, which avoided futile random search by diffusion/rotation *via* the increase of local concentration. A bivalent construct containing TNFR1 extracellular domain attached to an Fc domain was found to be more effective than the monovalent isolated extracellular domains (12). Furthermore, superior binding of the TNFR1-Fc inhibitor to TNF than to an anti-TNF mAb was demonstrated (12). In a more recent study it was demonstrated that the TNFR2-Fc fusion product known as etanercept was able to bind in an intramolecular fashion to TNF (41). The authors demonstrated that two discrete, relatively small soluble complexes were formed by the interaction of etanercept to the cytokine, whereas two different anti-TNF mAbs generated extremely large complexes as the result of the avid interaction of both Fab arms to two different TNF molecules, which could be visualized as precipitates in ouchterlony assays.

In contrast, in a cellular system, the mAb infliximab was a superior and more stable inhibitor of membrane-bound TNF than a TNFR-Fc inhibitor, despite comparable avidities (42). This was explained by the ability of the mAb to attach to a higher number of TNF trimers at the cell surface; the TNFR-Fc inhibitor can bind the two sites only *via* the intramolecular interaction with two of the three receptor interaction sites of the cytokine, while the mAb is able to saturate the three sites present on the cell surface, but using the two Fab arms to bind to two different cytokine molecules expressed on the surface of the cell.

Herein, bivalent/bispecific Nanobodies™ imbedding a sufficiently long linker were shown to bind with high avidity to TNF, occupying two of its three sites known to interact with TNFR (Figures 4A,B). Contrary to previously reported mAbs (41, 42), the very flexible geometry of the linker and the small size of the Nanobody™ binding site (as compared to a conventional antibody, where the binding site consists of the VH and VL) make it possible for these bivalent constructs to bind the two receptor interaction sites of the cytokine. When saturation occurs, it may be possible to obtain a complex of five bivalent/bispecific Nanobodies™ on two TNF molecules. On the other hand, under non-saturating conditions a single TNF molecule may be bound by two bivalent Nanobody™ constructs, which thereby occupy all three receptor binding sites. With a large excess of cytokine over bivalent Nanobody™ one can speculate that only a single Nanobody™ molecule will bind the TNF trimer, thereby leaving one free receptor binding site; this complex, when interaction with a single receptor molecule will block the cross-linking of this receptor molecule and might therefore function as an antagonist. When the linker is too short, preventing association of the second Nanobody™ module on the same TNF, high order networks of Nanobody™ bivalent/bispecific Nanobody–TNF complexes will form as was observed for anti-TNF mAb, which comes with a lower potency of neutralization (41).

Interestingly, a Tg197 transgenic human TNF mouse model for polyarthritis, confirmed *in vivo* the validity of properly designed Nanobody™ bivalent constructs against arthritis. For this purpose, the TNF Nanobodies™ were combined with the anti-HSA Nanobody™ (which cross-reacts with mouse serum albumin) to achieve appropriate serum half-lives. Bivalent VHH#3 alone or in combination with VHH#1 Nanobody™ in a bispecific construct displayed the best binding to TNF. Their efficacy *in vivo* was found to be comparable to that of the receptor-based inhibitor TNFR-Fc etanercept. Topological considerations indicated that both the 9 amino acid linker of VHH#3-9GS-VHH#3-9GS-HSA and the ~30 amino acid (9GS-HSA-9GS) linker of VHH#1-9GS-HSA-9GS-VHH#1 (taking into account that the albumin-specific Nanobody™ spans a length of approximately 12 amino acids) provide distances compatible with the binding of both VHH#3 and VHH#1 modules to the same TNF molecule. This finding is key to explaining the excellent *in vivo* efficacy of these bivalent Nanobody™ constructs.

## Ethics Statement

This study was carried out in accordance with the recommendations of Ministry of Research, Belgium.

## Author Contributions

EB: leading program, supervisor research *in vitro* and *in vivo* studies; writing of the manuscript. AD: performing structural studies. SS: crystallized the complexes, collected and integrated the X-ray data. ML: performing experimental work on nanobody production, formatting into multimeric constructs, and purification of complexes for structural studies. LA: bioassay measurements. TD: designing and supervising *in vivo* efficacy studies. CC: solved, refined the structures and wrote the paper. RL: interpretation of structural data and writing of sections on structural studies. KS: involved in experimental work and writing of the manuscript. CP: supervising the research, corresponding author. HH: inventor of and performed the first series of formatting experiments, initiated structural studies, and manuscript writing.

## Conflict of Interest Statement

EB, ML, TD, KS, CP, and HH: Ablynx employee during this research. AD, SS, LA, and CC: research paid in part by contract with Ablynx. RL: no conflict of interest.
